# A dynamic Brugada sign due to left anterior descending coronary artery occlusion

**DOI:** 10.1016/j.ipej.2022.09.003

**Published:** 2022-10-01

**Authors:** Vickram Vignesh Rangaswamy, Aparna Velmurugan, Akshay Balaji, S. Balaji

**Affiliations:** Department of Cardiology, Sri Ramakrishna Hospital, Coimbatore, India

**Keywords:** Brugada phenocopy, Brugada syndrome, Brugada sign, Acute myocardial infarction, Acute coronary syndrome

## Abstract

Brugada phenocopies (BrP) include several conditions with a common electrocardiographic (ECG) pattern that are indistinguishable from classical Brugada syndrome (BrS). In this report, we describe two cases of acute myocardial infarction (AMI) presenting as BrP. The majority of cases of BrP in AMI have been reported due to right coronary artery (RCA) occlusion. Rarely, the left anterior descending artery (LAD) is incriminated as the cause. In both our cases of BrP, LAD was the culprit vessel.

## Highlights

• Acute anterior myocardial infarction due to LAD occlusion can present as Brugada phenocopy.
• Analysis of 12 lead ECG for ischemia in other leads apart from the Brugada sign can help identify a BrP.
• Application of clinical pre-test and diagnostic criteria helps differentiate a BrP from a BrS.

## 1 Introduction

Brugada phenocopies (BrP) are heterogenous clinical entities with Brugada-like ECG pattern. Among them, cardiac emergencies such as acute myocardial infarction, pulmonary embolism, and hyperkalaemia can masquerade as Brugada like ECG pattern [1]. Rapid identification of these conditions in the emergency room (ER) can increase the likelihood of survival. In this report, we describe a series of cases of acute coronary syndrome (ACS) with a Brugada-like ECG pattern (Brugada sign) and how careful analysis of standard 12-lead ECG helped in successful outcomes.

## 2 Case 1

A 46-year-old man presented to the cardiology clinic with complaints of atypical chest discomfort on and off for 3 days. His cardiovascular risk factors include type 2 diabetes mellitus, systemic hypertension, and family history of coronary artery disease (CAD). There was no family history of arrhythmia or sudden cardiac arrest (SCA). His clinical examination was unremarkable. His initial ECG showed sinus rhythm with horizontal ST segment depression with T wave inversion in the anterior leads (Fig. 1A). Transthoracic echocardiogram did not show regional wall motion abnormalities. Laboratory results, including blood troponin, were in the normal range. Patient was admitted and serial ECGs were performed. Subsequent ECG recorded during chest pain showed the emergence of Type 1 Brugada ST elevation (Fig. 1B). Despite the presence of type 1 Brugada pattern, other accompanying ECG signs were inconsistent with the diagnosis of BrS. As an result, diagnosis of ACS presenting as BrP was considered, and patient underwent coronary angiogram. It revealed critical stenosis of the proximal left anterior descending artery before the origin of diagonal and septal branches, and other coronary arteries were found to be normal. (Supplementary image 1.1) He successfully underwent percutaneous coronary intervention (PCI) to LAD. Post procedure ECGs showed resolution of the Brugada pattern and the emergence of an evolved myocardial infarction picture (Fig. 1C).

**Fig. 1:**
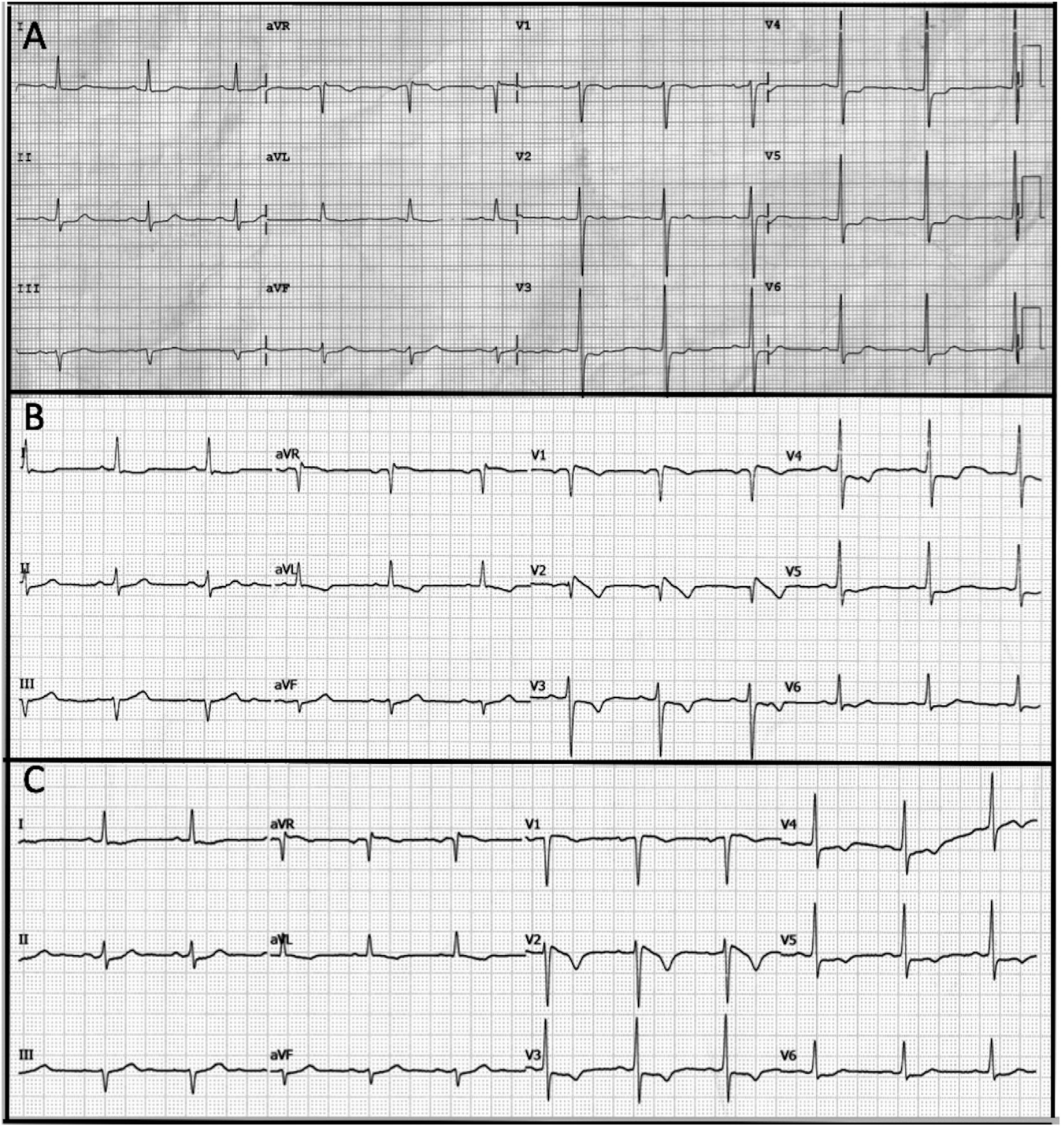
A: ECG taken at presentation showing horizontal ST segment depression with T wave inversion in anterior leads Fig. 1B: ECG taken during an episode of chest pain showing the emergence of Brugada sign in V1–V3 along with ST segment elevation (STE) in aVR and ST segment depression along with reversal of T wave polarity in V3–V5 and aVL. Fig. 1C: ECG taken post PCI to LAD vessel showed the resolution of Brugada sign.

## 3 Case 2

**Fig. 2:**
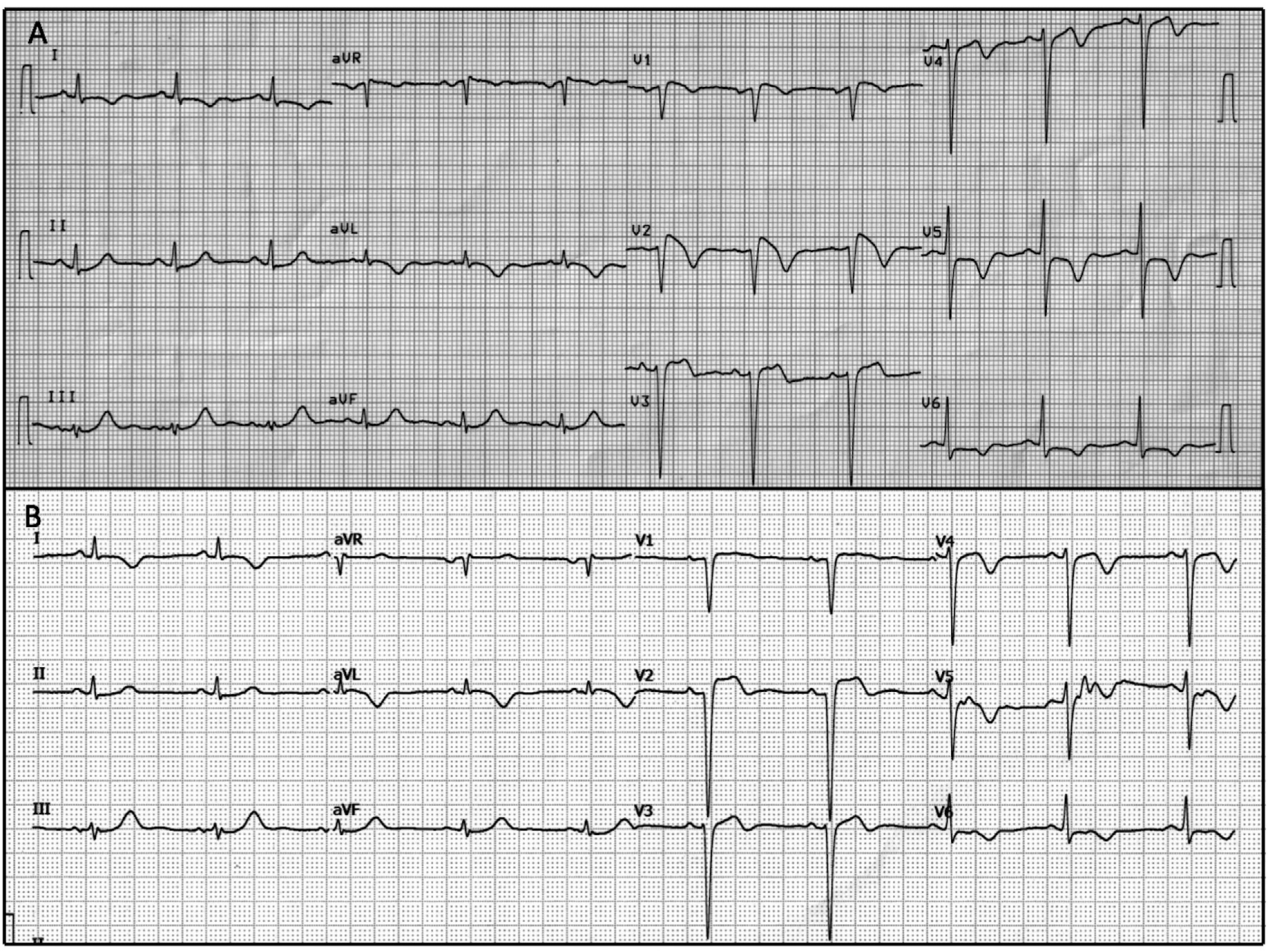
A: ECG taken during chest pain shows Brugada sign in V1–V2 and deep T wave inversion in anterolateral leads (V4-6 and I and aVL) Fig. 2B: ECG taken post PCI to LAD vessel showed resolution of ECG changes.

A 76-year-old male without any cardiovascular risk factors presented to the emergency room with acute onset retrosternal chest pain of 6 hour duration. There was no family history of arrhythmia or sudden cardiac arrest (SCA). His clinical examination was normal. His ECG revealed sinus rhythm with ST elevation mimicking type 1 Brugada pattern: 3 mm J point elevation with an coving ST segment elevation with a rapidly descending T wave inversion in lead V1 –V2 which is characteristic of Brugada sign (Fig. 2A). Deep T wave inversion was seen in anterolateral leads (V4-6 and I and aVL). His echocardiogram revealed mild hypokinesia of anterior wall segments. His blood troponin was elevated. Coronary angiogram revealed total thrombotic occlusion of LAD at ostioproximal segment (Fig. 2C) and a non-dominant RCA vessel. He underwent primary angioplasty with stenting to ostial-proximal LAD. (Supplementary image 1.2) Post procedure ECG showed coving ST elevation of the Brugada type replaced by a flat ST segment elevation (Fig. 2B).

## 4 Discussion

Congenital BrS is primarily an electrical disorder that predisposes to malignant ventricular arrhythmia and sudden cardiac death. BrS is diagnosed by a characteristic ST segment elevation pattern in lead V1-3. The pathogenesis of BrS is attributed to abnormal repolarization, and also to depolarization abnormalities within the right ventricle, especially the right ventricular outflow tract (RVOT) [1].

Apart from BrS, several other disorders can produce similar patterns and are listed under the umbrella term of BrP. It is equally important to recognize these entities as treatment is different for each one of them. In this report, we have described two cases of AMI that presented with Brugada sign, and an early diagnosis was possible due to high index of suspicion of MI and application of pre-test probability. The clinical pre-test probability includes the presence of clinical symptoms, medical history, and family history suggestive of BrS [2]. Both our cases were found to have a low clinical pre-test probability for BrS.

Myocardial infarction (MI) is a well-recognized cause of BrP [3]. Various coronary artery pathologies capable of producing ischemia such as critical coronary artery disease, vasospasm, coronary arteriovenous fistula, coronary slow flow, and balloon dilatation have precipitated BrP. In majority of these cases, the culprit artery was RCA followed by LAD and, Left circumflex artery (LCX) was the least involved artery [3]. In both our cases, LAD was the infarct related artery.

Ischemia of the right ventricle (RV), especially RVOT (by virtue of the conal branch supplying RVOT), has been implicated in the pathogenesis of the Brugada pattern. Ischemia induced sodium channel dysfunction and increased outward flow of potassium ions (Ito) in the RVOT epicardium, causing dispersion of phase 2 action can be a potential factor. Di Diego et al.*,*studied BrP caused by ischemia in a canine model with a perfused RV wedge and compared it to BrS. They found similar operating mechanisms in both the groups for ST elevation by virtue of accentuation of AP dome in some areas and prolongation of the R wave in other areas [4]. In humans, there seems to be no specific association between the BrP and RV ischemia, as BrP has been reported in all the three coronary artery territory occlusions.

Some authors advocate a sodium channel provocation test to differentiate between a BrP from a true BrS [5]. Unlike in BrS, sodium channel blockers don't produce dynamic ECG changes in AMI, as BrP is due to the transient nature of ion channel dysfunction and treatment of ischemia normalizes the BrP. This can help to distinguish between a BrP secondary to an ischemic cause and rare cases of ischemia unmasking a BrS [6]. Although advocated, provocative testing can be risky in the peri-infarct period. Routine screening of family members can also help with differentiation. In our cases, genetic screening was not done due to its low diagnostic yield, as SCN5A mutation is identifiable in only 20–30% of the patients.

A clear differentiation between the BrS and the ACS induced BrP is important, as treatment is entirely different between the two scenarios. Occasionally, BrS has been misdiagnosed as MI and even subjected to thrombolysis [7]. Similar to BrS, myocardial ischemia can present as sudden cardiac arrest due to ventricular fibrillation (VF) during active ischemia. Further, troponin positivity in patients with resuscitated cardiac arrest due to BrS delays the diagnosis. Any event that causes a delays in diagnosis of MI during the golden hour will result in a critical loss of myocardium and invariably result in a delay in the door to balloon time. It is also possible for true BrS get unmasked by ischemia. Van Malderen et al. have reported a case of ischemia and increased vagal tone precipitating ventricular fibrillation in BrS [8].

Certain criteria have been proposed to differentiate BrP and BrS in these settings. These criteria are Brugada type ECG pattern, identifiable underlying condition, resolution of ECG pattern, low clinical pre-test probability, negative provocative sodium channel blocker test, and genetic testing [9]. Both our cases, met 4 out of the 6 mentioned criteria to qualify as BrP. The clinical pre-test probability is a tool that helps to assess and differentiate between a BrP and a BrS, especially in states of diagnostic confusion when an acute MI unmasks a BrS.

## 5 Conclusion

The recognition of ischemia induced BrP as a differential diagnosis of BrS is significant, as the prognosis and treatment outcomes are essentially different. The analysis of the 12-lead ECG should be done meticulously to look for other ECG signs of alternate aetiology in the presence of a Brugada type pattern.

## Declaration of interests

The authors declare that they have no known competing financial interests or personal relationships that could have appeared to influence the work reported in this paper.

## Figures and Tables

**figs1:**
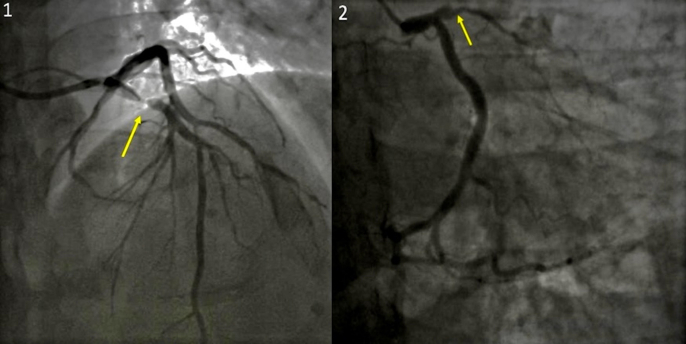


## Abbreviations

AMI –: Acute Myocardial Infarction
ACS –: Acute coronary syndrome
BrP –: Brugada phenocopy
BrS –: Brugada syndrome
CAD: Coronary Artery Disease
ECG –: Electrocardiography
ER –: Emergency room
LAD –: Left anterior descending
LCX –: Left circumflex artery
PCI –: Percutaneous coronary intervention
RVOT –: Right ventricular outflow tract
RV –: Right ventricle
RCA –: Right coronary artery
STE –: ST segment Elevation
VF –: Ventricular fibrillation

## Appendix A Supplementary data

Supplementary image 1.1: Coronary angiogram showed a 90% stenosis in proximal LAD before the origin of diagonal and septal branches.

Supplementary image 1.2: Coronary angiogram showing complete occlusion of proximal LAD.
